# *Salmonella* Typhimurium in Kolkata: Evidence of Intra-familial Transmission

**Published:** 2007-03

**Authors:** A. Palit, S. Dutta, D. Sur, M.K. Bhattacharya, S.K. Bhattacharya

**Affiliations:** ^1^ Department of Microbiology; ^2^ Department of Epidemiology; ^3^ Department of Clinical Medicine, National Institute of Cholera and Enteric Diseases (Indian Council of Medical Research), P-33, C.I.T. Road, Scheme XM, Beliaghata, Kolkata 700 010, West Bengal, India

Sir,

Salmonellosis has been recognized as one of the major public-health problems in many parts of the world, and *Salmonella enterica* serovar Typhimurium is the commonest serotype encountered in most geographical situations (1). Endemicity of *Salmonella* has been shown to be maintained through a cyclic order from man to the environment and vice-versa (2). Explosive outbreak due to a common-source contamination is rather a rare event, and person-to-person transmission has been believed to be operating in the spread of *S. enterica* serovar Typhimurium (3–5).

Symptomatic and asymptomatic persons in the community usually transmit salmonellosis. However, the exact mode of its transmission has not so far been elucidated. The present study was an attempt to determine the modes of transmission of *S. enterica* serovar Typhimurium in the endemic community of Kolkata, West Bengal, India.

The study was carried out in a periurban slum of Kolkata (Tiljala) during August 2002–July 2003. In total, 610 stool cultures clinically suspected to have diarrhoeagenic *Salmonella* without any prior antibiotic therapy in the preceding five days were included in the study.

Stool samples were collected and inoculated in buffer peptone water, followed by enrichment in selenite F or tetrathionate broth (Difco Laboratories Ltd., Maryland, USA. Stool cultures were incubated for 24 hours in each culture and were then subcultured on selective media (Difco Laboratories Ltd.). The suspected *S. enterica* serovar Typhimurium colonies were identified as per the protocol (6) and were serologically confirmed using commercially-available antisera from Denka Seikan (Japan).

Various sources of water examined were ponds, tubewells, tap water (piped), and open wells and also containers. About one litre of water was collected in sterilized bottles directly from the sources. All the water samples were transferred to a laboratory within 2–3 hours and were filtered through Millipore filter membrane of pore size 0.22 μm (Millipore Corporation, USA). The filtered membranes were inoculated in the same broth as mentioned earlier. The pH of water from various sources was determined.

Eight (1.3%) of the 610 stool samples were positive for *S. enterica* serovar Typhimurium, of which most interestingly three were from the same family. No other enteropathogens could be detected from these three samples. Of these three, one was an index-positive case (the first case to be officially reported from the focus), and two were isolated from household contacts of the same family.

Detailed interviews revealed that stored pond water from an earthen trough was used for washing the mouth by both index subject and other two subjects infected, approximately 20 hours before the onset and subsequent acuteness of symptoms. This fact accords with the known incubation period of *S. enterica* serovar Typhimurium. Contaminated improperly-washed hands of the index case may have spread it, through which faecal matter with *S. enterica* serovar Typhimurium might have gone in the trough water, and subsequent usage by the other two persons facilitated its further transmission through the faecal-oral route.

Detection of *S. enterica* serovar Typhimurium in different categories of water samples is presented in the Table. Three (4.0% approximately) of 76 water samples collected were positive for *S. enterica* serovar Typhimurium. Of these, two were isolated from the pond-water samples, and one was from the stored water sample from the household of the index case. The pH of water varied from 8.2 to 8.6 (alkaline in nature).

**Table.** Isolation of Salmonella enterica serovar Typhimurium from various sources of water samples

Source of water samples examined	No. of water samples examined	Positive cases	
No.	%
Pond	10	2	20
Open well	14	-	
Stored	45	1	2.2
Tubewell	5	-	
Tap	2	-	
Total	76	3	4.0

*S. enterica* serovar Typhimurium-associated infection in all the three family members was likely to be transmitted through consumption of contaminated milk which was also supported by the detection of positive culture of *S. enterica* serovar Typhimurium obtained from leftover milk samples in milk-containers collected from the house of the subjects. Milk-containers are washed with pond-water (which was invariably contaminated) (Table). Thereby it was inferred that *Salmonella* must have contaminated the containers/milk contact surfaces after their washing with the contaminated pond-water prior to use.

It may, thus, be postulated that simple provision of safe water in the community may be a single important intervening factor in controlling the spread of infection, as can be inferred from the isolation of *S. enterica* serovar Typhimurium from stored water and pond-water. However, continuous piped water supply within the houses is of utmost importance to arrest the contamination point, otherwise people will have no other options but to store water in an unhygienic condition or use pond-water, thereby leading to sustained transmission of the infection. Consumption of stored water and usage of pond-water due to lack of sufficient piped water are the most likely ‘risk factors’ for the sporadic occurrence of *S. enterica* serovar Typhimurium-associated infection among the infected subjects.

Rapid identification of the possible source of transmission and prompt implementation of control measures can curtail the spread of this sort of infection. Intra-familial transmission of *Salmonella* may explain a large proportion of cases of paediatric salmonellosis. Older children and adults with salmonellosis may be the most important source of infection for infants.
